# Challenges and breakthroughs: current landscape and future prospects of CAR-T cell therapy clinical trials for solid tumors

**DOI:** 10.3389/fonc.2025.1652329

**Published:** 2025-09-03

**Authors:** Jing Guo, Chunhe Zhou, Hongmei Zhao, Huiyan Li

**Affiliations:** The Affiliated Cancer Hospital, Harbin Medical University, Harbin, Heilongjiang, China

**Keywords:** CAR-T cell therapy, clinical trials, solid tumors, tumor microenvironment, antigen escape

## Abstract

Chimeric antigen receptor T-cell (CAR-T) therapy has demonstrated significant efficacy in the treatment of hematological malignancies; however, its application in the solid tumor setting remains challenging. Given that solid tumors account for the vast majority of clinically diagnosed cancers, there is an urgent and significant clinical need to develop effective CAR-T therapy. This review focuses on the latest clinical trials of CAR-T therapy in major solid tumors, including glioma, colorectal, pancreatic, prostate, and lung cancers. It systematically evaluates the results of studies targeting key tumor-associated antigens, such as EGFR, IL13Rα2, GD2, B7-H3, CEA, MSLN, PSCA/PSMA, and ROR1. The results indicate that locally delivered, dual-targeted CAR-T cells and engineered CAR-T cells show potential in reducing antigenic escape and enhancing cellular function. Significant survival benefit and tumor remission were observed in some studies. However, antigen heterogeneity-driven escape, tumor immunosuppressive microenvironment, insufficient persistence of CAR-T cells *in vivo*, and treatment-related toxicity still limit their efficacy and clinical application. To address these challenges, we further discuss various optimization strategies, including target selection, combination of immune checkpoint inhibitors or tumor microenvironment modulators, and optimization of CAR structural design and delivery methods. In the future, through the exploration of multi-dimensional optimization design and combination therapeutic regimen, it is expected to facilitate the broader application and clinical translation of CAR-T therapy in solid tumor treatment.

## 1 Background

Chimeric antigen receptor T-cell (CAR-T) therapy is a new approach to tumor immunotherapy. It involves genetically engineering T cells to express a receptor (CAR) that specifically recognizes tumor antigens, thereby activating T-cell cytotoxic function. This technology has made significant progress in the treatment of hematological malignancies: 12 CAR-T products have been approved worldwide (1), with complete remission rates ranging from 60% to 90% for relapsed/refractory B-cell leukemia (2), lymphoma, and multiple myeloma (MM). However, solid tumors account for about 90% of clinical cancers (3), and CAR-T therapy is significantly less effective in treating solid tumors than hematologic tumors. This is due to physical barriers in solid tissues that limit T-cell infiltration, an immunosuppressive microenvironment that weakens cellular function, and tumor antigen heterogeneity promoting antigen escape. For highly lethal solid tumors such as glioblastoma (GBM) and pancreatic cancer, researchers are actively leveraging successful strategies from hematological malignancies to promote the clinical translation of CAR-T therapy for solid tumors. Efforts focus on exploring novel tumor-associated antigenic targets and optimizing CAR structure design and innovative delivery strategies. Early clinical trial results have confirmed the feasibility of these strategies, laying important groundwork for further enhancing efficacy and optimizing safety.

## 2 Introduction

Current CAR-T therapies approved by global regulatory agencies primarily target the B-cell antigen CD19 or the B-cell maturation antigen (BCMA). Tisagenlecleucel (Kymriah^®^), the world’s first CAR-T product, was approved by the FDA in 2017 to treat relapsed or refractory acute lymphoblastic leukemia (ALL) (4). Obecabtagene autoleucel (Aucabtzyl^®^, 2024), the first CD19 CAR-T therapy exempt from REMS requirements, is also approved for R/R ALL (5). For lymphoma, axicabtagene ciloleucel (Yescarta^®^, 2017) (6) and lisocabtagene maraleucel (Breyanzi^®^, 2021) (7) are approved to treat large B-cell lymphoma (LBCL). Brexucabtagene autoleucel (Tecartus^®^, 2020) is the first CD19 CAR-T therapy approved for mantle cell lymphoma treatment (8). In addition to CD19 targets, CAR-T therapies targeting BCMA have also been approved. Idecabtagene vicleucel (Abecma^®^, 2021) was the world’s first approved BCMA CAR-T therapy for MM (9); ciltacabtagene autoleucel (Carvykti^®^, 2022) (10); and China’s self-developed BCMA CAR-T therapies were approved in 2023–2024 (11). Together, these advances are reshaping the treatment landscape for hematologic malignancies. CD19-targeted therapies have significantly improved survival outcomes for patients with B-cell ALL and aggressive B-cell lymphomas. BCMA CAR-T therapies provide durable remission in patients with MM, representing a significant shift in the treatment paradigm of this disease.

However, despite the significant success of CAR-T therapy in treating hematological malignancies, it still faces multiple challenges in treating solid tumors. The primary obstacle is the highly immunosuppressive tumor microenvironment (TME), which has dense physical barriers, abnormal vasculature, and an abundance of immunosuppressive cells and factors. These features collectively hinder the effective infiltration of CAR-T cells, significantly inhibiting their activation, proliferation, and persistence. Ultimately, this leads to functional exhaustion. Additionally, the widespread heterogeneity of tumor antigen expression easily leads to target escape, resulting in incomplete treatment or recurrence. Furthermore, solid tumors lack ideal, tumor-specific target antigens. Most potential targets are also expressed in normal tissues, which significantly increases the risk of “off-target/off-tumor toxicity.” Overcoming these interconnected and complex obstacles is crucial for extending the revolutionary benefits of CAR-T therapy to a broader population of patients with solid tumors. This review systematically summarizes the progress of CAR-T clinical trials targeting major solid tumor types. It includes a section (Section 4.1) that focuses on common, core optimization strategies (such as target optimization, delivery technology innovation, TME regulation, and functional enhancement design) across different tumor types. This section more clearly outlines the key pathways and future directions for CAR-T development in solid tumors.

To improve the readability of the terminology, abbreviations are used in this article as shown in **Table 1**. Key terms are marked with their full names when they first appear and are subsequently referred to using abbreviations.

**Table 1:** Core abbreviation index.

Abbr.	Full Form
CAR-T	Chimeric antigen receptor T-cell
CAR	T cells to express a receptor
MM	multiple myeloma
GBM	glioblastoma
BCMA	B-cell maturation antigen
ALL	acute lymphoblastic leukemia
LBCL	large B-cell lymphoma
TME	tumor microenvironment
CRC	colorectal cancer
IV	intravenous
ICIs	immune checkpoint inhibitors
DIPG	Diffuse intrinsic pontine glioma
ICV	intracerebroventricular
ORR	objective response rate
PFS	progression-free survival
OS	overall survival
TRAEs	transient treatment-related adverse events
CRS	cytokine release syndrome
DLTs	dose-limiting toxicities
CEA	Carcinoembryonic antigen
SD	stable disease
GCC	guanylate cyclase C
PDAC	Pancreatic ductal adenocarcinoma
MSLN	mesothelin
HER2	human epidermal growth factor receptor 2
PSCA	prostate stem cell antigen
EGFR	epidermal growth factor receptor
PCs	pancreatic cancers
mPDAC	metastatic pancreatic ductal adenocarcinoma
PSMA	prostate-specific membrane antigen
mCRPC	metastatic castration-resistant prostate cancer
NSCLC	non-small cell lung cancer
ROR1	receptor tyrosine kinase-like orphan receptor 1
MPM	malignant pleural mesothelioma
HCC	hepatocellular carcinoma
VEGF	vascular endothelial growth factor
sVEGFR2	soluble VEGF receptor 2
SDF-1	stromal cell-derived factor-1
EPC	endothelial progenitor cell

## 3 Methods

The following search terms were used in the literature search for related articles: “CAR-T cell”, “glioblastoma”, “GBM”, “colorectal cancer”, “pancreatic cancer”, “prostate cancer”, “lung cancer”, “NSCLC”, “breast cancer”, “ovarian cancer”, “mesothelioma”, “MPM”, “hepatocellular carcinoma”, “HCC”, “kidney cancer”. Searches were conducted on PubMed and Embase from inception to May 2025. A total of ten searches were conducted on each database: “CAR-T cell” and “glioblastoma” or “GBM”, “CAR-T cell” and “colorectal cancer”, “CAR-T cell” and “pancreatic cancer”, “CAR-T cell” and “prostate cancer”, “CAR-T cell” and “lung cancer” or “NSCLC”, “CAR-T cell” and “breast cancer”, “CAR-T cell” and “ovarian cancer”, “CAR-T cell” and “mesothelioma” or “MPM”, “CAR-T cell” and “hepatocellular carcinoma” or “HCC”, “CAR-T cell” and “kidney cancer”. The inclusion criteria encompassed clinical trials of CAR-T therapy for solid tumors, while studies on hematological malignancies, non-CAR-T therapies, and conference abstracts lacking full data availability were excluded.

## 4 Results

### 4.1 Overview of core strategies for CAR-T therapy across solid tumor types

Despite the differences in the biological characteristics, anatomical locations, and target antigen expression among various solid tumors, the development of CAR-T therapies is centered on several common core optimization strategies. This section will synthesize research examples from subsequent chapters (4.2 - 4.11) involving different tumors to outline key optimization directions in solid tumor CAR-T therapy and conduct preliminary cross-tumor comparisons.

Core Strategies:

- Target Antigen Selection and Design Optimization: To overcome antigen heterogeneity and escape, researchers are actively developing dual-/multi-target CAR-T cells and logic-gated CARs. Examples include EGFR/IL13Rα2 CAR-T for GBM, CD19/GCC “Coupled CAR” for colorectal cancer (CRC), and TAG - 72/ΔCD47m CAR for ovarian cancer. Ongoing exploration of novel or optimized targets includes B7-H3 and GD2 for brain cancer, MSLN and CEA for pancreatic cancer, MSLN and FAP for mesothelioma, and GPC3 for liver cancer. Escape phenomena caused by antigen heterogeneity are common across tumor types. Examples include the loss of EGFRvIII in brain cancer and recurrence after single-target therapy in pancreatic cancer. Additionally, differences in target distribution across normal tissues result in varying off-target toxicity risk profiles. For example, HER2-targeting requires vigilance for cardiotoxicity and pulmonary toxicity, while CEA-targeting warrants attention to intestinal toxicity.
- Innovations in Delivery Routes: Localized delivery strategies, such as intracranial injection for brain cancer, intrapleural injection for mesothelioma, and intratumoral injection for breast cancer, are widely employed. These routes aim to enhance CAR-T concentration at the tumor site, overcome physical barriers, and reduce systemic toxicity. Repeated infusion strategies of GD2 CAR-T and B7-H3 CAR-T are also used to address the issue of insufficient persistence in brain cancer. Comparing the advantages, disadvantages, and challenges of different delivery methods is crucial. For example, intracranial injection of GD2 CAR-T in brain cancer has demonstrated significantly prolonged patient survival, highlighting its advantage in overcoming the blood-brain barrier. In contrast, intravenous (IV) infusion in colorectal and lung cancers frequently encounters challenges of insufficient persistence and poor tumor infiltration. Intratumoral injection of GD2 CAR-T in breast cancer has been shown to induce local necrosis and immune infiltration but lacks systemic efficacy. Furthermore, various local delivery approaches each face challenges, including procedural complexity, feasibility of repeated administration, and site-specific risks such as intracranial hemorrhage and injection-site infections.
- Overcoming TME Suppression: Key strategies include: (1) Engineering CAR-T cells to secrete immunomodulatory factors, such as pancreatic cancer MSLN-CAR-T cells that secrete IL - 7/CCL19 (“7×19”); (2) Combining CAR-T therapy with immune checkpoint inhibitors (ICIs), such as MSLN-targeted CAR-T combined with pembrolizumab in mesothelioma, with future plans to explore combinations with PD - 1 inhibitors in brain and lung cancers; (3) Combining CAR-T with TME modulators, including potential combinations with TGF-β inhibitors in prostate cancer research, and considerations for future combinations with anti-angiogenic agents in liver and kidney cancers. The degree of immunosuppression within the solid tumor TME varies across tumor types. For instance, pancreatic cancer and GBM are generally considered archetypes of highly immunosuppressive TMEs, which may partially explain the relatively weaker CAR-T therapy efficacy. In contrast, mesothelioma combined with PD - 1 inhibitors has yielded more promising results.
- Enhancing CAR-T Cell Function and Persistence: Optimizing co-stimulatory domain combinations is a key focus. For instance, research on CRC has clearly demonstrated that second-generation CAR constructs containing a CD28 domain outperformed third-generation designs incorporating a CD137 domain *in vitro*. Another important focus is developing integrated safety switches, such as the EGFR safety switch in ovarian cancer MUC16 CAR-T. Furthermore, exploring universal CAR-T cells represents a significant direction, exemplified by CD70 CAR-T in kidney cancer. Insufficient cellular persistence also constitutes a universal core challenge. Reported persistence data vary across tumor types but are universally limited: prostate cancer shows median persistence of less than 14 days, CRC is maintained for four to six weeks, and ovarian cancer declines below the detection limit within one month.

Subsequent sections (4.2 - 4.11) will detail specific clinical trial data by tumor type, collectively outlining the current landscape and future optimization strategies for CAR-T therapy in solid tumors.

### 4.2 Brain cancer

GBM and H3K27M-mutant diffuse intrinsic pontine glioma (DIPG) are important areas of exploration for CAR-T cell therapy due to their high aggressiveness and extremely poor prognosis with conventional treatments. Several phase I clinical trials targeting EGFR, IL13Rα2, GD2, and B7-H3 have initially demonstrated the immune activation potential and manageable toxicity of such therapy through localized delivery strategies such as intrathecal or intracranial administration.

In a single-arm, open-label phase I trial (n = 18), researchers first used an intracerebroventricular (ICV) injection of dual-targeted EGFR/IL13Rα2 CAR-T cells to treat recurrent GBM(NCT05168423). Among 13 patients with measurable disease, tumor regression was observed in 8 (62%). One patient achieved a confirmed partial response, resulting in an objective response rate (ORR) of 8%. Notably, significant CAR-T cell expansion signals were detected in the patients’ cerebrospinal fluid. The median progression-free survival (PFS) was 1.9 months (90% CI, 1.1–3.4 months), and the median overall survival (OS) had not yet been reached at the data cutoff point (median follow-up time of 8.1 months). In terms of safety, 56% of patients experienced grade 3 neurotoxicity, which occurred across all dose groups (12). It should be noted that the single-arm, non-randomized design of this study may not establish a causal relationship between efficacy and treatment, and the open-label design may introduce detection bias.

Another single-arm, open-label phase I trial (n=10) evaluated IV infusion of single-target EGFRvIII CAR-T cells to treat GBM (NCT02209376). Despite significant downregulation of target antigen occurred in tumor tissue in five out of seven patients after surgery, upregulation of PD-L1 and IDO1 expression was concurrently observed in the TME. This suggests a possible association with treatment resistance induction. Since most patients underwent neurosurgical intervention, PFS could not be assessed, and the median OS was 251 days. Although no objective response (ORR = 0%) was observed, 90% of patients achieved short-term disease stabilization (13). However, the heterogeneity of EGFRvIII expression (6–96%) and inconsistent timing of surgery may limit the generalizability of the conclusions. Similarly, a single-arm phase I trial (n=3) used intracranial local infusion of CD8^+^ CAR-T cells targeting IL13Rα2 to treat GBM (NCT00730613). This trial was designed for post-operative treatment. PFS data were not reported and ORR assessment was not feasible; only transient anti-tumor activity was observed. Post-recurrence survival times were 10.3, 8.6, and 13.9 months, respectively. Two patients experienced grade 3 or higher transient treatment-related adverse events (TRAEs). Notably, the study failed to stratify enrollment or analysis based on IL13Rα2 expression levels (14). Consequently, pooling patients with high and low expression may have obscured potential correlations with efficacy.

For pediatric lethally progressive DIPG, a single-arm phase I trial (n=11) compared the IV versus ICV administration routes for GD2-CAR T cells (NCT04196413). Results indicated that the IV DL2 group (3 × 10^6^ cells/kg) was limited by 37.5% grade 4 cytokine release syndrome (CRS), leading to the designation of the DL1 dose (1 × 10^6^ cells/kg) as the maximum tolerated dose. Conversely, ICV therapy resulted in tumor regression in 7 patients (ORR = 63.6%), including one achieving complete remission sustained for over 30 months. The median OS was 20.6 months for all patients, and no dose-limiting toxicities (DLTs) were observed with the ICV route (15). However, the study included individualized treatment differences that may interfere with efficacy analysis. Another single-arm, open-label Phase I trial (n=21) evaluated repeated ICV infusions of B7-H3-targeted CAR T cells in patients with DIPG, achieving a median OS of 19.8 months (NCT04185038). Among patients with prior progression, 88.9% achieved disease control, with an ORR of 6%. Three patients survived for over 44 months. Regarding safety, only one grade 4 intracranial hemorrhage event was reported (16). Furthermore, elevated inflammatory factor levels in the cerebrospinal fluid confirmed local immune activation. Nevertheless, significant differences in disease status and time to diagnosis were observed at the time of patient enrollment, which may have introduced uncontrollable confounding biases.

Taken together, these trial results reveal that the core challenges impeding current CAR-T therapy for brain tumors include susceptibility to antigen escape with single-target approaches, such as loss of EGFRvIII, and an immunosuppressive TME (e.g., upregulation of PD-L1/IDO1), and insufficient CAR-T cell persistence. Therefore, future strategies will focus on: advancing combination therapy of dual-target (EGFR/IL13Rα2) CAR-T cells and PD-1 inhibitors for GBM; exploring dual targeting of GD2/B7-H3 or combination with radiotherapy for DIPG; and enhancing T cell activity through repeated intracranial infusion and incorporation of 4 - 1BB co-stimulatory domains, while integrating ctDNA monitoring and AI-based imaging for precise assessment. These integrated approaches aim to propel CAR-T therapy beyond achieving safety and controllability towards inducing durable remissions.

### 4.3 Colorectal cancer

Carcinoembryonic antigen (CEA) is highly expressed in the majority of patients with CRC (98.8%) (17), making it an important target for CAR-T cell therapy. A single-arm, open-label phase I dose-escalation trial (n=10) evaluated a CEA-targeted CAR T-cell therapy (NCT02349724). The therapy was well tolerated. Following CAR-T treatment, seven patients (70%) achieved stable disease (SD), and two cases of SD persisted for more than 30 weeks. However, no objective response was achieved (ORR = 0%). And two others showed tumor shrinkage via PET/CT and MRI analyses. Most patients showed a significant decrease in serum CEA levels during long-term observation. However, CAR-T cells persisted *in vivo* for only four to six weeks before rapidly decaying to undetectable levels, which suggests limited persistence of the cells (18). Given its therapeutic limitations and preclinical studies confirming that the second-generation CAR structure containing the CD28 co-stimulatory domain outperforms the third-generation design containing CD137 *in vitro* experiments. The authors suggest in their discussion that future research could explore the following directions: optimizing co-stimulatory signal combinations; incorporating chemokine receptors to enhance tumor infiltration; and combining with ICIs to overcome TME suppression.

Another single-arm phase I trial (n=15) explored a dual-target strategy. Using the “Coupled CAR” platform, the study targeted CD19 and guanylate cyclase C (GCC) to treat patients with multilineage resistance (ChiCTR2000040645). Among these, 6 patients achieved partial response (ORR = 40%). At the time of data cutoff, the median OS was 22.8 months (95% CI: 13.4 – 26.1). The PFS in the high-dose group (2 × 10^6^ cells/kg) was 6.0 months (95% CI, 3.0 to not available), and in the low-dose group (1 × 10^6^ cells/kg) it was 1.9 months (95% CI, 1.0 to not available). However, 14 out of 15 patients (93%) experienced at least one grade 3 or higher adverse event. Additionally, all patients reported grade 1 – 2 cytokine release syndrome, and one patient experienced grade 4 neurotoxicity (19). These results suggest the need for individualized dose adjustments such as step-up strategies and prophylactic toxicity management measures such as anti-inflammatory therapy and supportive care. While the results demonstrate the potential synergy of dual-target strategies, their mechanisms of action require further elucidation in multicenter randomized trials.

Two single-arm phase I trials (combined n=16) evaluated first-generation retroviral-transduced CAR-T cells (CART72) targeting tumor-associated glycoprotein (TAG - 72) in patients with metastatic CRC. Ten patients were treated in trial C - 9701 and six in trial C - 9702. No objective response was observed in either trial; all patients had progressive disease. In the C - 9702 trial, one patient experienced two transient decreases in CEA levels, potentially indicative of cytokine release syndrome. Despite a favorable safety profile, the majority of patients developed anti-idiotype antibodies against CC49, which led to rapid clearance of subsequent CAR-T cell infusions (20). Additionally, the trials were conducted prior to modern CAR-T standards, and toxicity assessment used the early CTCAE v2.0 standard, limiting comparability with current studies.

In summary, CAR-T cell therapy for CRC faces three core challenges: limited cell persistence, tumor microenvironment-mediated immunosuppression, and immune escape due to antigenic heterogeneity. Optimization strategies focus on three aspects: enhancing CAR-T function through enhanced T-cell activation signaling, improving tumor homing ability, and multi-target design; modulating the immunosuppressive TME through combination with ICIs or chemotherapy; and reducing immune rejection through all-humanization technology. Future research should rely on large-scale randomized trials to verify efficacy, incorporate dynamic immune monitoring to explore optimal timing for early intervention, and focus on developing both off-the-shelf products and individualized approaches to overcome therapeutic limitations in CRC.

### 4.4 Pancreatic cancer

Pancreatic ductal adenocarcinoma (PDAC) accounts for over 90% of pancreatic cancer cases. Its highly aggressive and immunosuppressive microenvironment results in an extremely poor prognosis, with a five-year survival rate of less than 10% (21). Although immunotherapies such as PD - 1/PD-L1 inhibitors are limited in their efficacy, more targeted CAR-T cell therapies have emerged as an important research direction. Progress has been made in phase I clinical trials targeting mesothelin (MSLN), human epidermal growth factor receptor 2 (HER2), prostate stem cell antigen (PSCA), and epidermal growth factor receptor (EGFR).

Among PDAC-related targets, MSLN has been the most extensively studied. Approximately 80% of PDAC patients’ tumor tissues express MSLN, rendering it a promising target with broad application potential (NCT03198546). In a single-arm, open-label Phase I trial (n=6, including 1 case of PDAC), investigators developed “7×19” modified CAR-T cells, a therapy that enhances T-cell infiltration and survival in the TME by secreting IL - 7 and CCL19. Preliminary results showed that one patient with advanced PDAC achieved complete response in hilar lymph node metastases, as assessed by CT, without experiencing grade ≥ 3 CRS or neurotoxicity (22). However, due to the trial’s small sample size and limited follow-up duration, PFS and OS were not reported, the efficacy and safety of this therapy require validation in larger studies.

HER2 is a potential target in pancreatic cancers (PCs). A single-arm, open-label phase I trial (n=11, including 2 PCs) showed that both pancreatic cancer patients achieved SD (ORR = 0%), with PFS durations of 5.3 and 8.3 months, respectively (23). However, due to the low level of HER2 expression in normal gastrointestinal tissues and the risk of off-target toxicity of this therapy, as well as the limited persistence of CAR-T cells *in vivo*, the clinical benefit of this therapy needs to be confirmed by conducting larger studies (NCT01935843). On the other hand, the study was characterized by a small sample size and a highly heterogeneous population (encompassing biliary tract cancer and pancreatic cancer), potentially compromising the ability to reliably evaluate efficacy differences or identify predictive biomarkers.

PSCA is expressed in approximately 50% of patients with metastatic pancreatic ductal adenocarcinoma (mPDAC). A single-arm, open-label phase I trial (n=20) evaluated the BPX - 601 CAR-T therapy targeting PSCA (NCT02744287). None of the 20 patients with mPDAC achieved complete remission or partial remission, and no PFS or OS data were reported, only three exhibited tumor burden reduction. Additionally, the main DLTs were hematologic toxicities, and the incidence of CRS was 12.5%. Notably, the trial was terminated early due to two cases of dose-limiting toxicity, including one case of fatal sepsis, in the highest dose group of other cancer types, and the dose exploration was not completed (24).

CAR-T therapy targeting EGFR, an important target in metastatic pancreatic cancer, has demonstrated some clinical efficacy. In a single-arm, open-label phase I trial (n=14), 4 evaluable patients achieved PR for 2 – 4 months (ORR = 28.6%) (NCT01869166). The median OS was 4.9 months (range, 2.9 – 30 months), and median PFS was 3 months (range: 2 – 4 months). Although some patients experienced reversible grade ≥ 3 adverse events, including fever/fatigue, nausea/vomiting, and mucocutaneous toxicity (25). A limitation is that all patients received chemotherapy-based lymphodepletion conditioning regimens, making it difficult to exclude the contribution of these interventions to tumor response, thereby weakening the reliability of attributing CAR-T therapy efficacy. Another single-arm, open-label phase Ib trial (n=1) reported one case of pancreatic adenocarcinoma with liver metastases who achieved complete metabolic response as assessed by PET-CT after receiving CEA-targeted CAR-T therapy. (OS: 23.2 months) (26). Unfortunately, the tumor recurred and did not respond to subsequent treatment (NCT02850536). The core limitation lies in the severely insufficient sample size, which prevents statistical inference.

In summary, CAR-T cell therapy shows potential in PDAC treatment but still faces core challenges, including immunosuppression in the tumor microenvironment, insufficient CAR-T cell persistence, off-target toxicity, and tumor antigen heterogeneity. Future breakthroughs necessitate integrated multidimensional strategies: Technically, exploring dual-target designs such as EGFR/HER2, CEA/MSLN and advancing universal CAR-T cell development could enhance therapeutic precision and accessibility. Therapeutically, combining microenvironment modulators such as ICIs, TGF-β signaling pathway inhibitors or local radiotherapy may help overcome immunosuppressive barriers. At the clinical translation level, it is necessary to accelerate the advancement of rigorously designed, adequately powered, and standardized multicenter phase II/III clinical trials to validate long-term efficacy, and actively explore the translational potential of emerging targets, with the aim of providing pancreatic cancer patients with more effective treatment options.

### 4.5 Prostate cancer

A single-arm, open-label phase I clinical trial (n=14) targeting high expression of PSCA evaluated the safety and early efficacy of PSCA-CAR T cells in patients with metastatic castration-resistant prostate cancer. Results demonstrated that no patients achieved objective response (ORR = 0%). The SD rate was 67% (4/6) in dose level 2 (standard lymphodepletion) and 60% (3/5) in DL3 (reduced-intensity lymphodepletion). PFS and OS were not reported. Among the 14 treated patients, 5 experienced Grade 1 or 2 CRS (36%) (27). A key limitation was the omission of planned correlative analyses, including circulating tumor DNA sequencing, circulating tumor cell gene expression profiling, and CAR immunogenicity assessment, which compromised the study’s translational value.

Meanwhile, another single-arm, open-label phase I clinical trial (n=5) targeting prostate-specific membrane antigen (PSMA) (BB-IND #12084) evaluated the safety and pharmacodynamics of anti-PSMA engineered CAR-T cells combined with low-dose IL - 2 in patients with metastatic castration-resistant prostate cancer (mCRPC). Results showed that all patients achieved CAR-T cell engraftment (5 – 56%), with 2 patients achieving partial response (ORR = 40%). PFS and OS were not reported. There were no treatment-related deaths or anti-PSMA-targeted toxicities. IL - 2-related toxicities (fatigue/rash) were grade 1 – 2 (28). A key limitation was the use of a fixed low dose of IL - 2 without dynamic adjustment based on CAR-T expansion dynamics, leading to IL - 2 depletion in patients with high engraftment (>50%), which may result in suppression of effector T cell function. Additionally, the development of MRI-TRUS fusion robotic navigation systems provides a technological basis for the precise, intraprostatic injection of therapy, such as CAR-T cells. This approach could serve as an alternative to conventional local therapies such as high-intensity focused ultrasound, potentially enhancing drug concentration at the tumor site while reducing systemic toxicity (29).

In another study exploring strategies to overcome the immunosuppressive microenvironment, a single-arm, open-label phase I clinical trial (n=13) targeting mCRPC utilized PSMA as its target and integrated a dominant-negative TGFβ receptor (TGFβRDN) (NCT03089203). Results showed that no patients achieved objective response (ORR = 0%). Four patients achieved a ≥30% decline in prostate-specific antigen, and five had stable disease (SD: 38.5%). Median PFS was 4.4 months, and OS was 15.9 months. CRS of grade ≥2 occurred in 5 of 13 treated patients (38.5%), including two grade 3 events and one fatal event (grade 4) (30). It is important to note that more than 50% of patients received novel therapies after progression, and the OS (15.9 months) may be confounded by the contribution of subsequent therapies, making it difficult to solely reflect the efficacy of CAR-T therapy.

Together, these studies highlight three core challenges of CAR-T therapy in prostate cancer: antigenic heterogeneity, dynamic upregulation of immunosuppressive factors within the tumor microenvironment, and risks of dose-limiting toxicity. Addressing these challenges requires multidimensional optimization, such as developing multi-targeted CAR designs -combining PSCA with PSMA or integrating STEAP1-to overcome antigenic escape and remodeling immunosuppression by blocking TGF-β signaling or combining microenvironmental modulators. Individualized dosing is implemented based on CAR-T pharmacokinetic/pharmacodynamic models and combined with local delivery technologies to enhance tumor infiltration and reduce toxicity. Future efforts should integrate these strategies in expanded cohort studies to advance CAR-T therapy toward later-stage clinical translation, such as Phase III clinical trials, and establish more definitive efficacy.

### 4.6 Lung cancer

A phase I, single-arm, open-label trial evaluated autologous CAR-T cell therapy targeting receptor tyrosine kinase-like orphan receptor 1 (ROR1) in patients with advanced chronic lymphocytic leukemia and solid tumors (n=21) (NCT02706392). The cohort included 8 patients with non-small cell lung cancer (NSCLC). Safety for NSCLC was not reported separately. Among all patients, 22.2% experienced grade 3 CRS, and there was one dose-limiting grade 4 pulmonary toxicity-related fatal event. Pathological analysis confirmed the presence of CAR-T cell infiltration in the lungs and the high expression of PD - 1 and TIM - 3 immune checkpoint molecules by the infiltrating CAR-T cells. The remaining patients primarily experienced reversible grade 3 – 4 pulmonary adverse events, including dyspnea, hypoxemia, pulmonary edema, and pleural effusion. Regarding efficacy, no objective responses were observed (ORR = 0%); only transient disease stabilization occurred. Its limitation lies in the fact that the ROR1 expression threshold in the cohort is not standardized for intensity, which may include patients with insufficient antigen expression, thereby diluting the therapeutic effect (31). Therefore, to overcome these limitations, future strategies should focus on optimizing the CAR construct to incorporate immune checkpoint blockade functionality, adopting intensified lymphodepletion regimens to overcome these limitations.

Another single-arm, open-label phase I trial (n=9) used a non-viral piggyBac transposon system to construct EGFR-targeted CAR-T cells for the treatment of patients with advanced NSCLC expressing high levels of EGFR (NCT03182816). Patients received incremental infusions of cells at two dose levels over two cycles. The safety profile was favorable, with no serious cytokine release syndrome reported, and only grade 1 – 3 pyrexia events reported. CAR-T cells exhibited a rapid peak-to-attenuation pattern in peripheral blood. One patient achieved a partial response lasting 13 months (ORR = 11.1%). The median PFS for the entire group was 7.13 months (95% CI: 2.71 - 17.10 months), and the median OS was 15.63 months (95% CI: 8.82 - 22.03 months) (32). However, the study had obvious limitations, including a small sample size and inclusion of different pathological subtypes (7 adenocarcinomas and 2 squamous cell carcinomas) mean that observed efficacy differences may be confounded by underlying tumor biology. Subsequent recommendations include expanding the sample size and conducting stratified randomized trials, coupled with pre- and post-treatment tissue biopsies to deeply investigate TME inhibitory mechanisms.

### 4.7 Breast cancer

The hepatocyte growth factor receptor (c-Met) is a potential target for solid tumor therapy due to its high expression in approximately 50% of breast cancers and low expression in normal tissues (33). A single-arm, open-label, phase 0 clinical trial evaluated the intratumoral injection of c-Met-CAR-T cells modified by mRNA electroporation in six patients with metastatic breast cancer, four of whom had triple-negative breast cancer and two of whom had hormone receptor-positive cancer (NCT01837602). The results showed a favorable safety profile, with only mild local erythema and no cytokine release syndrome (34). Although no systemic tumor remission was observed, the injection site exhibited a decrease in c-Met expression, extensive necrosis, and immune cell infiltration, confirming local biological activity. This phase 0 trial assessed efficacy solely based on local pathological findings and did not report PFS or OS, making it impossible to evaluate systemic antitumor activity or the status of distant metastases. A subsequent phase I study treated seven patients with metastatic solid tumors (four with breast cancer and three with melanoma) with an IV infusion of autologous c-Met-CAR-T cells modified by RNA electroporation (NCT03060356). The regimen’s safety profile was similarly manageable;with grade 1 or 2 adverse events observed in all breast cancer patients. PFS range: 0.6 – 17 months; OS range: 1.3 – 24.7 months. However, efficacy remained limited, with only two breast cancer patients achieving stable disease (ORR = 0%) and no CAR-T cell infiltration detected in the tumor microenvironment (35). It should be noted that the small sample size of this study and the restriction of the primary efficacy assessment to day 25 (± 5 days) may fail to capture delayed-onset responses or pseudo-progression patterns characteristic of immunotherapy.

A comprehensive analysis revealed that c-Met-targeted CAR-T therapy faces several challenges. First, mRNA vectors resulted in CAR expression lasting fewer than seven days, which severely limited *in vivo* cellular persistence. Second, c-Met expression heterogeneity within tumors makes them prone to antigen escape. Third, physical and biological barriers impede tumor infiltration via the IV infusion route. To overcome these challenges, future efforts should combine biomarker screening (such as ctDNA) to identify patients with high c-Met expression and optimize the treatment window. These strategies should be validated in rigorously designed follow-up studies, including dose escalation, lymph node clearance, and multi-time point assessments.

### 4.8 Ovarian cancer

CAR-T cell therapy for ovarian cancer is still in the early stages of clinical exploration. The first single-arm, open-label Phase I clinical trial, which targeted the FRα antigen, enrolled 14 patients with metastatic ovarian cancer in 2006. The trial used MOV-γ CAR-T cells, which were infused at doses up to 5×10^10^. The study confirmed that the treatment’s overall safety. However, five patients in cohort 1 experienced grade 3 – 4 IL - 2–related toxicity, while cohort 2 only experienced grade 1 – 2 adverse events. None of the patients achieved tumor remission. The main issues identified included: insufficient persistence of CAR-T cells after infusion, which dropped below the detection limit within one month; inefficient tumor homing, with only one patient showing weak signals in peritoneal metastases; and the development of neutralizing antibodies in the serum of 50% of subjects, which significantly impaired T-cell function (36). In 2012, researchers proposed an improved FRα CAR-T cell therapy regimen (incorporating a 4 - 1BB co-stimulatory domain) for recurrent ovarian cancer and designed a single-arm phase I trial (n ≤ 15). The protocol employed strategies such as fractionated dosing, dose escalation, and infusion of untransduced peripheral blood lymphocytes to optimize safety. However, this publication solely describes the preliminary trial design and reports no clinical safety outcomes (37). While baseline FRα expression served as an enrollment criterion, the protocol failed to mandate FRα re-testing in tumors during or after treatment. This omission precluded assessment of the potential for antigen loss. Meanwhile, therapies targeting the MUC16 antigen successfully inhibited tumor growth in a mouse model by modifying the CAR to target its extracellular domain. A CAR-T therapy based on this target that combines an IL - 12 secretion module with an EGFR safety switch entered phase I clinical trials (38).

Furthermore, a preclinical study developed a novel dual-targeting CAR-T cell therapy for ovarian cancer. This approach fused a full-length CAR targeting TAG - 72 with a monomerically modified truncated CD47 CAR (ΔCD47m), aiming to overcome the dual challenges of antigen escape and off-target toxicity in solid tumor treatment. Experiments confirmed that dual-targeting CAR-T cells incorporating a 4 - 1BB co-stimulatory domain achieved a 60% kill rate against chemotherapy-resistant ovarian cancer models with low TAG - 72 expression. *In vivo* models demonstrated effective inhibition of tumor growth, with tumor volume reduced by 70% compared to the untreated control group (39). However, a primary limitation of this study is that immunodeficient mouse models cannot replicate the human microenvironment, necessitating further validation in large animal models and preclinical trials.

To achieve future breakthrough in CAR-T therapy for ovarian cancer, target selection should circumvent antigenic structural domains prone to shedding. Additionally, developing CARs targeting the MUC16 transmembrane domain or FRα/MUC16 tandem CARs may help overcome tumor heterogeneity and mitigate antigen loss. To enhance CAR-T infiltration into peritoneal metastases, local intraperitoneal infusion combined with hyperthermic intraperitoneal chemotherapy is being explored. To respond to the immunosuppressive microenvironment, a combination approach with ICIs or antibody-coupled drugs is necessary. Other strategies to improve the durability and safety of CAR-T cells *in vivo* include optimizing CAR structural design, such as utilizing humanized single-chain variable fragments to reduce immunogenicity and refining co-stimulatory domains.

### 4.9 Mesothelioma

Therapeutic studies for malignant pleural mesothelioma (MPM) are actively exploring new immunological strategies. MSLN is an ideal therapeutic target due to its nearly 100% expression rate in MPM and its correlation with tumor aggressiveness. A single-arm, open-label, phase I study evaluated the regional infusion of MSLN -targeted CAR-T cells combined with the PD - 1 inhibitor pembrolizumab in patients with MPM (NCT02414269). Eighteen MPM patients received the combination therapy, achieving a median OS of 23.9 months (95% CI: 14.7 months - NE) and a one-year OS rate of 83% (95% CI: 68%-100% months). Investigators proposed that pembrolizumab may synergistically enhance overall antitumor efficacy by activating endogenous T-cell responses and inducing antitumor antibody production (40). The variability in the timing of pembrolizumab administration (ranging from 4 to 17 weeks) introduces temporal confounding factors, potentially complicating the accurate assessment of synergistic mechanisms in the combination therapy.

In parallel, strategies targeting fibroblast activation protein (FAP) are also being explored. The phase I FAPME trial (n=3) first evaluated the safety of intrapleural infusion of FAP-targeted CAR-T cells for MPM treatment (NCT01722149). It is important to emphasize that FAP is widely expressed in the stroma of MPM tumors but virtually absent in normal tissues. Following a single low-dose intrapleural injection, three patients exhibited PFS of 12 months, 9 months, and ≥18 months, respectively. No grade ≥3 TRAEs were reported, and no ORR or OS data were reported. Interestingly, CAR-T cell expansion and elevated levels of proinflammatory factors were detected in the peripheral blood of one patient, suggesting that local treatment may have induced systemic immune activation. However, due to the limited sample size, the statistical power for assessing safety and efficacy was insufficient, and the study was susceptible to interference from outliers. Therefore, the antitumor efficacy of this study remains unclear (41). Subsequent studies are needed to expand the patient cohort, optimize the dose-escalation regimen, explore combinations with ICIs, improve local delivery technology to prolong CAR-T cell retention in the pleural cavity, and analyze FAP function in the TME to develop FAP-targeted therapeutic strategies.

### 4.10 Hepatocellular carcinoma

CAR-T cell therapy for hepatocellular carcinoma (HCC) has targeted multiple antigens, with significant progress made in exploring GPC3, CD133, and CD147. However, there is still room for optimization. A single-arm, open-label, phase I clinical trial targeting GPC3 demonstrated that a patient with advanced HCC who received a single intratumoral injection of 7×19 CAR-T cells targeting GPC3 experienced tumor shrinkage within 10 days and achieved complete imaging remission by day 32, with no grade ≥3 adverse events reported (22). The limitation lies in the severely insufficient sample size, precluding statistical inference (NCT03198546). In a single-arm, open-label, phase I/II trial, 21 patients with advanced HCC received CAR-T-CD133 cell therapy (NCT02541370). The median OS was 12 months (95% CI: 9.3 – 15.3 months), and the median PFS was 6.8 months (95% CI: 4.3 – 8.4 months). Of the 21 evaluable patients, 1 achieved a partial response, 14 had stable disease (lasting 2 - 16.3 months), and 6 experienced disease progression after T-cell infusion. The most common high-grade adverse event was hyperbilirubinemia. Clinical outcomes correlated with baseline levels of vascular endothelial growth factor (VEGF), soluble VEGF receptor 2 (sVEGFR2), stromal cell-derived factor-1 (SDF - 1), and endothelial progenitor cell (EPC) counts. Furthermore, post-infusion changes in EPC counts, VEGF, SDF - 1, sVEGFR2, and interferon-γ levels were associated with survival (42). It is important to note that there is significant clinical heterogeneity among enrolled patients. For example, patients with Child-Pugh grade B (57.1%) have poorer prognoses, which may dilute the treatment effect. Additionally, the high proportion of patients with prior treatment resistance may also impact the assessment of CART - 133 response.

Another preclinical study developed Dox-inducible CD147 CAR-T (Tet-CD147CAR) for HCC therapy. Through the Tet-On 3G system, the expression of CD147CAR can be precisely regulated by Dox. *In vitro* experiments demonstrated that Dox-induced CAR-T cells significantly enhanced the proliferation, cytotoxicity, and cytokine secretion against CD147^+^ tumor cells. *In vivo*, intratumoral injection of Dox-induced CAR-T cells significantly suppressed tumor growth in nude mice (43). Key preclinical limitations include the use of immunodeficient nude mouse models, which fail to recapitulate the human immune microenvironment, and the small experimental group size (n=6/group), resulting in limited statistical power.

Based on the above progress, future optimization should establish a dynamic biomarker monitoring system, such as monitoring target expression levels, changes in vascular endothelial growth factors, and immune cell dynamics, to guide personalized treatment. By conducting rigorously designed multicenter randomized controlled trials to elevate the level of evidence, and by optimizing CAR structural modifications (such as incorporating co-stimulatory domains or developing dual-targeting CARs), combined with ICIs or anti-angiogenic drugs to enhance synergistic effects, we aim to achieve a clinical breakthrough in the application of CAR-T therapy for the treatment of HCC.

### 4.11 Kidney cancer

In CAR-T therapy research for renal cell carcinoma, two clinical trials are exploring different optimization pathways that focus on the CD70 and CAIX targets, respectively. The CD70 study employed CRISPR technology to develop universal allogeneic CAR-T cells. This process involved inserting an anti-CD70 CAR expression cassette into the TRAC motif and disrupting the β2M gene to eliminate MHC class I expression. Additionally, the CD70 gene was disrupted to mitigate self-inflicted killing, thereby enhancing the antitumor efficacy of the T cells. In this single-arm, open-label, phase I trial involving 16 patients, 13 (81.3%) achieved disease control, comprising 12 patients with stable disease and 1 patient (6%) with a complete response (ORR = 6.3%). Notably, patients who achieved complete response remained relapse-free at three years. The median PFS was 2.9 months (95% CI: 1.7 - 6.0 months), and the median OS was 20.5 months (95% CI: 14.3 months - NA) (44). However, researchers may introduce assessment bias by evaluating RECIST efficacy locally rather than through independent central review (NCT04438083).

In contrast, a single-arm, open-label, phase I clinical trial of CAIX-targeted CAR-T cells(n=12) demonstrated that pretreatment with an anti-CAIX monoclonal antibody completely eliminated hepatobiliary toxicity. This enabled the safe escalation of the CAR-T cell dose to 2 × 10^9^. The median OS was 9.5 months in the combined cohorts without antibody pretreatment (Cohorts 1 - 2) and 12.5 months in the cohort receiving antibody pretreatment (Cohort 3). However, the full text did not explicitly report PFS data, and this strategy failed to induce objective clinical response (ORR = 0%) (45). Additionally, spanning eight years, this study occurred during a period of significant evolution in the standard care for metastatic renal cell carcinoma. Comparisons of survival outcomes between cohorts may be driven by treatment-era bias rather than the intervention itself. Nevertheless, it provides a paradigm for the proactive prevention and control of targeted toxicity in solid tumor CAR-T therapy.

To enhance anti-tumor activity, future optimization of co-stimulatory signaling domains is needed, as well as rigorous screening of tumor-specific antigens to reduce off-target risk. Combining lymphocyte clearance regimens to promote CAR-T expansion *in vivo* is also necessary. While the two studies approached optimization from different angles—antigen specificity refinement CAIX and CAR structural enhancement CD70—they collectively point towards a direction of synergistic multi-strategy combination. Specifically, the CD70 study confirmed the clinical feasibility of allogeneic universal CAR-T cells in RCC, although their efficacy, particularly durability, requires further improvement.

Based on the above research, **Table 2** systematically summarizes the key clinical and toxicity results of CAR-T therapy for major solid tumors, providing a basis for horizontal comparison.

**Table 2:** Key CAR-T clinical trials in solid tumors: target antigens, delivery routes, efficacy, and toxicity.

Targets	Disease	Drug administration routes	DCR	ORR	Toxicity criteria	Principal toxicities
EGFR、IL-13Rα2	glioblastoma	ICV	69%	8%	ASTCT	CRS:100% (G ≤ 2)
EGFRvIII	glioblastoma	IV	90%	0%	CTCAE	neurologic events:30%(G ≤ 2)
IL13Rα2	recurrent glioblastoma	Intracavitary delivery	NR	NR	CTCAE	headache:33.3%(G≥3)
GD2	H3K27M-mutant diffuse intrinsic pontine glioma	IV	27.3%	0%	CTCAE	CRS:37.5% (G≥4)
GD2	H3K27M-mutant diffuse intrinsic pontine glioma	ICV	88.9%	44.4%	CTCAE	CRS:33.9%
B7-H3	diffuse intrinsic pontine glioma	ICV	88.9%	6%	CTCAE	headache:81%
CEA	metastatic colorectal cancer	IV	70%	0%	CTCAE	fever:20% (G ≤ 2)
CD19、 GCC	metastatic colorectal cancer	IV	73%	40%	CTCAE	CRS:93% (G ≤ 2)
TAG-72	colorectal cancer	IV、Intra-HA	NR	0%	CTCAE	CRS:70% (G ≤ 2)
MSLN	pancreatic carcinoma	IV	100%	100%	CTCAE	CRS:0%
HER2	pancreatic carcinoma	IV	100%	0%	CTCAE	CRS:0%
PSCA	metastatic pancreatic	IV	60%	0%	ASTCT	CRS:12.5%
EGFR	metastatic pancreatic carcinoma	IV	85.7%	28.6%	CTCAE	fever:6% (G≥3)
CEA	metastatic pancreatic carcinoma	Hepatic artery infusion	100%	100%	CTCAE	CRS:0%
PSCA	metastatic castration-resistant prostate cancer	IV	50%	0%	CTCAE	CRS:36% (G ≤ 2)
PSMA	metastatic or recurrent prostate cancer	IV	40%	40%	CTCAE	NR
PSMA	metastatic castration-resistant prostate cancer	IV	38.5%	0%	ASTCT	CRS:23.1%(G≥3)
ROR1	non–small cell lung cancer	IV	NSR	NSR	ASTCT	NSR
EGFR	non–small cell lung cancer	IV	77.8%	11.1%	CTCAE	fever:77.8% (G ≤ 3)
c-Met	metastatic breast cancer	Intratumoral injection	16.7%	0%	CTCAE	erythema:50%(G = 1)
c-Met	metastatic triple-negative breast cancer	IV	50%	0%	CTCAE	NSR
α-FR	metastatic ovarian cancer	IV	0%	0%	CTCAE	hypotension、 dyspnea:62.5%(G≥3)
mesothelin	malignant pleural mesothelioma	Intrapleural administration	68.8%	12.5%	CTCAE	CRS:0%
FAP	malignant pleural mesothelioma	Intrapleural infusion	NR	NR	CTCAE	CD4 lymphocytes reduced:66.7%(G ≤ 3)
CD133	hepatocellular carcinoma	IV	71.4%	4.8%	CTCAE	hyperbilirubinemia:19.0%(G≥3)
CD70	clear cell renal cell carcinoma	IV	81.3%	6.3%	ASTCT	CRS:50% (G ≤ 2)
CAIX	metastatic renal cell carcinoma	IV	NR	0%	CTCAE	transient liver enzyme disturbances: 33.3%(G≥3)

DCR, Disease Control Rate (Complete Response + Partial Response + Stable Disease); ORR, Objective Response Rate (Complete Response + Partial Response); ICV, Intracerebroventricular; Intra-HA, Intrahepatic Artery; NR, Not Reported (data unavailable in the source publication); NSR, Not Separately Reported (data were reported in aggregate or combined with other categories and could not be isolated for this specific group/toxicity); ASTCT, American Society for Transplantation and Cellular Therapy criteria; CTCAE, Common Terminology Criteria for Adverse Events; CRS, Cytokine Release Symptom.

## 5 Challenges and opportunities

**Figure 1:**
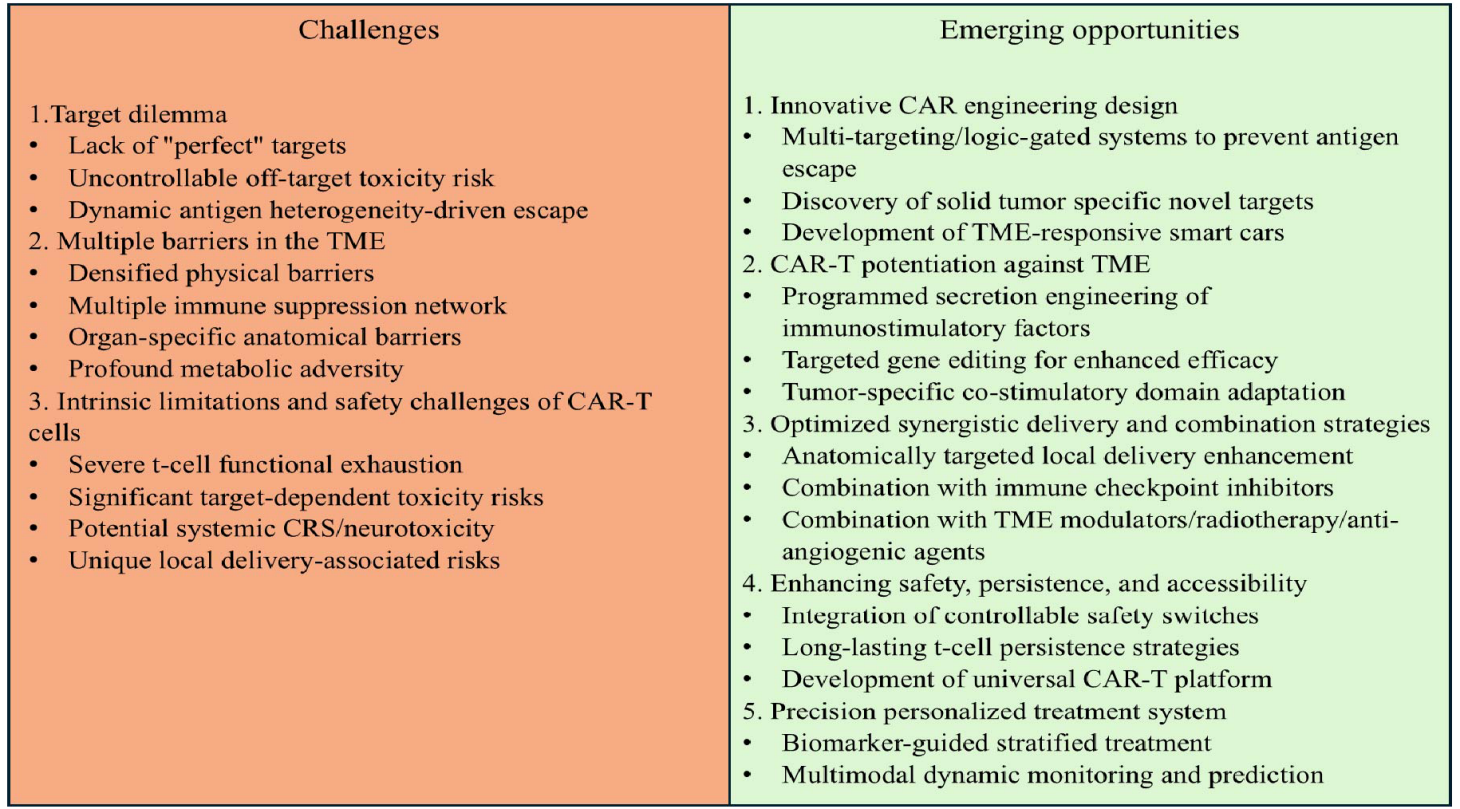
Key challenges and emerging strategies for CAR-T cell therapy in solid tumors.

As shown in **Figure 1**, in contrast to the remarkable success of CAR-T therapies in hematological malignancies, their application in the field of solid tumors faces a series of complex and interconnected obstacles, including tissue architecture, antigen expression patterns, and highly immunosuppressive tumor microenvironments, which severely limit the infiltration, activation, and persistence of CAR-T cells. However, continuous research innovations are opening up new pathways to overcome these barriers, with core strategies focusing on targeting optimization, microenvironment remodeling and delivery technology upgrades (**Figure 1**). Although CAR-T therapy has demonstrated significant efficacy in clinical trials for specific solid tumors (such as CEA-targeted colorectal cancer and GPC3-targeted hepatocellular carcinoma), its efficacy is inherently limited by the biological background of the tumor. The core limitation stems from the pronounced molecular heterogeneity observed among tumor subtypes within individual organs. Therefore, the efficacy observed in specific trials may not be generalizable to all histopathological or molecular subtypes of specific malignant tumors. The key to future progress lies in prioritizing the integration of deep molecular characterization analysis with precision oncology frameworks to enable biomarker-guided patient stratification and customized CAR-T strategies, bringing new hope to patients with solid tumors.

### 5.1 The target dilemma: heterogeneity, escape, and off-target toxicity

The primary challenge in CAR-T therapy for solid tumors is finding the ideal target antigen. Unlike hematological malignancies that have highly specific and uniformly expressed targets such as CD19 and BCMA, solid tumors generally lack such “perfect” targets (46). Tumor-associated antigens like MSLN, CEA, EGFR, ROR1, and c-Met are overexpressed in many solid tumors but often show low-level expression in normal tissues. This expression pattern poses a significant risk of off-target/on-target-off-tumor toxicity. For example, targeting HER2 requires caution regarding potential lung and cardiac toxicity; targeting CEA requires attention to intestinal toxicity; and targeting MSLN, B7-H3, GPC3, and other targets with low or limited expression in specific normal tissues may have different off-target risk profiles. This risk severely limits the therapeutic dose and the amount of treatment that can be delivered. This risk severely limits the therapeutic dose and window period. Another central challenge is antigenic heterogeneity. Significant differences in antigen expression within solid tumors and between different lesions result in single-target therapy being highly susceptible to failure due to antigen loss or down-regulation (47), making it possible that CAR-T cells, even if they reach the tumor site, may fail to efficiently eliminate all tumor cells, ultimately leading to disease progression.

### 5.2 Tumor microenvironment: multiple immunosuppressive barriers

The highly immunosuppressive TME constructed by solid tumors constitutes a major physical and biochemical barrier to CAR-T cell function. Dense extracellular matrix, abnormal vascular structure, and high interstitial pressure form a physical barrier that severely impedes CAR-T cell infiltration, often trapping them at the tumor periphery (48). The TME is enriched with immunosuppressive factors and cells, such as regulatory T cells, M2-type macrophages, and myeloid-derived suppressor cells. These components directly inhibit CAR-T cell activation, proliferation, effector function, and persistence. Furthermore, they induce T cell exhaustion through mechanisms such as nutrient depletion, secretion of inhibitory molecules, and expression of immune checkpoint molecules (49). In addition, physical barriers unique to the anatomical location of tumors (such as the blood-brain barrier in brain cancer and the peritoneum in ovarian cancer) are also key factors limiting the effective delivery of CAR-T cells to tumor cells and their ability to kill them, thereby affecting their efficacy. Harsh metabolic environments such as hypoxia, glucose deprivation, and lactate accumulation also further weaken the vitality and viability of CAR-T cells. Together, these factors lead to limited expansion, low functionality and insufficient persistence of CAR-T cells in the solid tumor microenvironment.

### 5.3 Intrinsic limitations and safety challenges of CAR-T cells

In addition to microenvironmental inhibition, CAR-T cells generally exhibit functional limitations in solid tumor patients. Advanced-stage patients frequently present with T cell exhaustion, senescence, and impaired baseline T cell function. The quality of the starting T cells, activation method, and cytokine cocktail may also affect the quality and *in vivo* activity of the final infused product. Meanwhile, the selection of co-stimulatory domains in the CAR structure has a significant impact on the activation strength, persistence and depletion tendency of T cells, which still needs to be further optimized (50). As found in CRC research (Section 4.3), CAR structures containing the CD28 domain outperform those containing the CD137 domain *in vitro*. This suggests that optimization of costimulatory domain selection must be tailored to the specific tumor type and overall CAR design.

Treatment-related toxicity still needs to be finely managed. Although the incidence of cytokine release syndrome and neurotoxicity may be lower in solid tumors than in hematological malignancies, the risk is significant in specific cases. Targeted/off-target toxicity is a significant safety concern specific to solid tumors. While localized delivery strategies can reduce systemic toxicity, they may also introduce distinct locoregional risks. For example, intracranial injection for glioma, intrapleural delivery for mesothelioma, intratumoral injection for breast cancer, and intraperitoneal infusion for ovarian cancer significantly reduce the incidence of severe CRS but introduce unique local risks, such as a reported case of grade 4 intracranial hemorrhage in brain cancer. Therefore, comparing the incidence and types of ≥3-grade adverse events reported across different delivery routes is crucial for optimizing safety design.

### 5.4 Innovative strategies and future directions

Facing significant challenges, researchers are developing multiple innovative strategies to enhance the efficacy of CAR-T therapy. At the CAR-T design level, dual-target or multi-target designs, as well as logic-gated CARs, are being employed to overcome heterogeneity and reduce antigen escape (51). Concurrently, researchers are actively exploring emerging targets and developing affinity-optimized CARs, programmable CARs, or “smart” CARs activated by the TME to enhance targeting precision and reduce off-target risks. While improving targeting precision, researchers are also focused on empowering CAR-T cells to counteract the immunosuppressive TME. To this end, CAR-T cells can be engineered to secrete immunostimulatory cytokines or express neutralizing molecules, which can directly improve their survival, recruitment, infiltration, and function within the microenvironment (52). Additionally, optimizing co-stimulatory domain selection and using gene editing technologies to modify T cells (such as knocking out inhibitory receptors or overexpressing anti-exhaustion factors) can enhance their vitality and resistance to exhaustion. Beyond cellular modifications, optimizing delivery and combination strategies is also critical. Local or regional delivery (such as intraperitoneal, intrathoracic, intratumoral, or intra-arterial) can significantly increase CAR-T cell concentration at the tumor site, reduce systemic toxicity, and overcome physical barriers. In terms of combination therapy, combining ICIs (such as PD-1 inhibitors) can help alleviate microenvironmental suppression, reverse T cell exhaustion, and activate endogenous immunity (53); while combining microenvironment modulators, radiotherapy, or anti-angiogenic drugs can reshape the ecological niche conducive to CAR-T function from multiple dimensions.

Enhancing safety, durability and accessibility are also key directions. Integration of suicide genes or drug-regulated switches can enable precise control of activity. Optimization of manufacturing processes to enrich stem cell-like memory T-cells to enhance *in vivo* durability and expansion. Development of universal CAR-T to overcome limitations such as long manufacturing time, high cost, and poor quality of patient T-cells in autologous products to improve accessibility. Use circulating tumor DNA to monitor tumor burden and clonal evolution, combine multiparametric imaging to assess tumor metabolism and immune infiltration, and apply artificial intelligence to integrate multi-omics data to perform dynamic assessment and prediction, and to guide individualized treatment.

In addition, achieving truly personalized CAR-T therapy necessitates careful consideration of patient age (children or adults). Children have more active and plastic immune systems, with superior T-cell proliferation potential and stemness, which favors CAR-T persistence. However, thymic activity can influence the T-cell receptor repertoire and CAR-T phenotype. In contrast, adults frequently face age-related immunosenescence, characterized by T-cell exhaustion and telomere shortening, which weakens CAR-T potency (54). Clinical considerations: The disease spectrum differs significantly: the primary pediatric indication for children is ALL, while adults encompass a broader range of conditions such as diffuse LBCL and MM (55). Toxicity profiles also exhibit distinct variations: pediatric patients typically exhibit stronger CRS inflammatory responses but better physiological compensatory capacity; adults are more susceptible to complications such as prolonged hematopoietic suppression and often have heavier disease burdens, leading to reduced tolerance. Crucially, long-term sequelae such as growth and development, neurocognitive function, and fertility preservation are unique core concerns for the pediatric population. Future innovative strategies must integrate age-stratified designs to enhance efficacy, safety management, and long-term monitoring frameworks for each population, driving the development of customized CAR-T therapies.

### 5.5 Integration pathway to clinical translation

Translating these promising innovations into tangible clinical benefits requires systematic integration: the development of CAR-T with multi-target recognition is only the starting point and must be combined with precision delivery and intelligent combination programs. In-depth analysis of the heterogeneity of the TME and the dynamic mechanism of CAR-T-microenvironment interactions is the basis for achieving precision therapy. Accelerating rigorous phase II/III randomized controlled trials to confirm survival benefit is critical. Optimization of general-purpose CAR-T manufacturing processes and cost reduction are key to widespread adoption (56). Exploring CAR-T application in earlier-stage disease or adjuvant/neoadjuvant settings represents an important strategy. In these settings, tumor burden is lower and immunosuppression is less pronounced, better facilitating CAR-T cell expansion, immune memory establishment, and micro metastasis eradication, thus enhancing efficacy. Through continued interdisciplinary innovation and collaboration, CAR-T therapy is expected to overcome these barriers and become an important force with transformative potential in the solid tumor treatment landscape.

## 6 Conclusion

The exploration of CAR-T cell therapy for solid tumors remains in the early stages of attack. This paper reviews the progress of the extensive clinical trials currently underway for a wide range of major solid tumors. Although most current studies are in early stages, they have preliminarily validated the therapy’s relative feasibility and safety. Furthermore, promising signals of anti-tumor activity, such as objective response or stable disease, have been observed in some patients. However, its overall level of efficacy still faces significant challenges. The inherent biological complexity of solid tumors, particularly antigenic heterogeneity, immunosuppressive microenvironment, and physical barriers, constitute the core obstacles limiting the effective infiltration, persistence, and functionality of CAR-T cells. The primary contribution of current research lies in the systematic identification of these critical bottlenecks and outlining potential avenues for breakthroughs, including innovative CAR designs, optimized delivery strategies, and multi-mechanism combination approaches. Future efforts must focus on continuous research innovation and rigorous clinical translation pathways to fully realize the potential of CAR-T therapy to revolutionize the solid tumor treatment landscape.
